# Roles of IL-11 in the regulation of bone metabolism

**DOI:** 10.3389/fendo.2023.1290130

**Published:** 2024-01-30

**Authors:** Yijing Han, Hui Gao, Xinling Gan, Jingying Liu, Chuncha Bao, Chengqi He

**Affiliations:** Department of Rehabilitation Medicine, West China Hospital, Sichuan University, Chengdu, Sichuan, China

**Keywords:** IL-11, bone, metabolism, immunity, systematic review

## Abstract

Bone metabolism is the basis for maintaining the normal physiological state of bone, and imbalance of bone metabolism can lead to a series of metabolic bone diseases. As a member of the IL-6 family, IL-11 acts primarily through the classical signaling pathway IL-11/Receptors, IL-11 (IL-11R)/Glycoprotein 130 (gp130). The regulatory role of IL-11 in bone metabolism has been found earlier, but mainly focuses on the effects on osteogenesis and osteoclasis. In recent years, more studies have focused on IL-11’s roles and related mechanisms in different bone metabolism activities. IL-11 regulates osteoblasts, osteoclasts, BM stromal cells, adipose tissue-derived mesenchymal stem cells, and chondrocytes. It’s involved in bone homeostasis, including osteogenesis, osteolysis, bone marrow (BM) hematopoiesis, BM adipogenesis, and bone metastasis. This review exams IL-11’s role in pathology and bone tissue, the cytokines and pathways that regulate IL-11 expression, and the feedback regulations of these pathways.

## 1 Introduction

Bone is in constant renewal and is a relatively dynamic organ compared with other organs (1). Bone metabolism is the basis for maintaining the normal physiological state of bone, mainly manifested as the coordination of bone resorption and bone formation (2). Imbalance of bone metabolism can lead to a series of metabolic bone diseases (3). The metabolism and remodeling of the bone is around 100% in the first years of life and is reduced to around 18% in adulthood, undergoes a progressive decrease, giving rise to conditions of osteoporosis. Osteoporosis, with the aging of the global population, will represent a global health problem in the future, and in this sense knowing all the mechanisms that contribute to its development is of fundamental importance for implementing preventive strategies. Bone cells interact with immune cells under physiological and pathological conditions (4). Therefore, immune-related cytokines play an important role in regulating bone metabolism. The interleukin family is an important part of pro-inflammatory cytokines. At present, IL-1, IL-4, IL-6, IL-17, IL-18 are the most explored interleukin factors in bone metabolism (5).

The pleotropic cytokine interleukin-11 (IL-11) exists mainly in a tetra helical structure and exerts its effects on hematopoietic cells. Human IL-11 cDNA encodes a 19-kDa protein containing 178 amino acids (6). IL-11, together with IL-27, IL-6, IL-31, leukemia inhibitory factor, oncostatin-M, cardiotrophin-1, cardiotrophin-like cytokine factor 1, and ciliary neurotrophic factor, belongs to the family of IL-6 cytokine that acts through the glycoprotein 130 (gp130) receptor subunit. IL-11 was initially detected in a primate bone marrow (BM) cell line and promotes plasmacytoma cell proliferation (7). It is expressed in several tissues and cells, including fibroblasts, mesenchymal stem cells, epithelial cells, hematopoietic cells, central nervous system neurons, osteoclasts, osteoblasts, adipocytes, synovial cells, chondrocytes, testis, and the gastrointestinal tract (8–12). IL-11 participates in several physiological processes, including bone remodeling, hematopoiesis, carcinogenesis, fertility (13), and nervous system pathologies (14).

IL-11Rα1 as well as IL-11Rα2 receptors showing transcytotic activity are localized in the basolateral and apical membranes of polarized epithelial cells. IL-11Rα1 shows expression in the brain, thymus, lungs, heart, spleen, kidneys, bladder, muscles, large and small intestines, BM, salivary glands, ovaries, uterus, and testes (15), and IL11Rα2 shows expression in the thymus, lymph nodes, and testes (16). After binding to IL-11R, a nonsignaling membrane receptor, IL-11 induces heterodimer formation in the signaling receptor gp130 (17–19) and stimulates the mitogen-activated protein kinase (MAPK) cascade and the Janus kinase/signal sensor and activator of transcription (JAK/STAT) pathway (20, 21). The IL-11/IL-11R complex formation differs from that of other cytokines in terms of thermodynamic and structural mechanisms (22). After binding to IL-11R1, IL-11 is transported through transcytosis and then secreted into the extracellular space (23). IL-11 is also implicated in a trans-signaling pathway as it binds to the soluble form of IL-11R (sIL-11R) and activate cells not expressing IL-11R (24–27). However, sIL-11R may antagonize IL-11 activity based on the amount of gp130 molecules in cells expressing transmembrane IL-11R (28).

As a member of the IL-6 family, the regulatory role of IL-11 in bone metabolism has been found earlier, but mainly focuses on the effects on osteogenesis and osteoclasis. In recent years, more studies have focused on IL-11 and its role and related mechanisms in different bone metabolism activities, and some studies have suggested that IL-11 can be used as a possible target for the treatment of bone diseases. This article will briefly summarize the role of IL-11 in inflammatory diseases, detail the regulatory role and related mechanisms of IL-11 in bone metabolism, and provide new ideas for the studies and treatment of metabolic bone diseases.

## 2 The regulatory role of IL-11 in inflammation

As a downstream gene of profibrotic factors, particularly TGF-β1 (29), IL-11 upregulation increases fibroblast proliferation and transformation (30), thereby leading to fibrosis and failure of tissues and organs (31–33). IL-11 is produced by lung stromal cells in response to virus infection and other factors. Furthermore, IL-11 upregulation in mouse airway cells triggers lymphocytic inflammation, which is characterized by fibroblast accumulation and increased collagen I and III expression (34). IL-11 modulates airway remodeling in individuals with asthma by promoting airway fibrosis and inhibiting alveolar development (35). IL-11Rα and IL-11 show a high expression in stellate as well as parenchymal cells in the pancreas and liver and induce inflammation, fibrosis, steatosis, and organ failure through secretion in an autocrine or paracrine manner (36–39), his is an important pathogenetic mechanism in alcoholic hepatitis and acute pancreatitis (40). IL-11 blocks gastric acid secretion in the gastrointestinal tract (41), leading to chronic gastroenteritis (42, 43). Hence, IL-11 could serve as a potential therapeutic target for inhibiting the TGF-β1/IL-11/MEK/ERK/mTOR and TGF-β1/Smad3/HIF-1α/IL-11 signaling pathways to prevent senescence-associated organ inflammation and fibrosis (44–48). IL-11Rα knockdown or deletion can also effectively prevent these diseases (30).

IL-11 exerts anti-inflammatory effects. Treatment with IL-11 decreases the expression of pro-inflammatory mediators such as IL-1β, IFN-γ, NF-κB, IL-6, TNF-α, and nitric oxide (49); reduces IL-12 production in macrophages; and increases the expression level of IL-4 and IL-10 (50). The IL-11-mediated regulation of the anti-inflammatory effect restores Th1 and Th2 cell balance as well as alleviates T cell-mediated liver injury (51–53). IL-11 prevents IFN-γ-induced death of hepatocytes by downregulating the IFN-γ/STAT1, JAK-STAT, MAPK, PI3K/AKT, and NF-κB signaling pathways (54). IL-11 reduces acute inflammation (55–57). Moreover, although IL-11 promotes airway fibrosis, it inhibits asthma-like inflammation (58, 59), and its expression is regulated by IL-13 (60, 61).

IL-11 also participates in tumor initiation and progression by regulating epithelial cell turnover (62) (9) and the IL-11/gp130/STAT3 signaling pathway. Neoplastic cell invasion and tumor growth were reduced following the pharmacological inhibition of IL-11/STAT3 in human tumor cell xenografts and mouse gastrointestinal cancer models (63).And IL-11 can alleviate cardiac fibrosis after myocardial infarction (64) and protects hepatocytes in liver ischemia through the activation of the IL-11/gp130/STAT3 signaling pathway (65).

## 3 Role of IL-11 in the regulation of bone metabolism

### 3.1 Bone formation

IL-11 regulates osteogenesis through several pathways. IL-11 signaling by membrane-bound receptors is a critical factor that allows craniofacial bones and teeth to develop normally in mice (66). Previous studies also confirmed this finding by demonstrating that mutations in IL-11 receptors induce abnormal bone development in mice (67–69). Fracture hematoma contains a large amount of inflammatory and immunomodulatory mediators. The high levels of anti-inflammatory molecules, including IL-11, in fracture hematoma induce extracellular matrix (ECM) production, angiogenesis, and osteogenesis (70). Quantitative PCR analysis showed the highest IL-11 mRNA expression level (increase by 73-fold, *p* < 0.0001) at 24 h following an ulnar stress fracture, thus indicating that IL-11 plays a key role in activating tissue remodeling and stress fracture healing (71). IL-11 induces osteoblast differentiation through the MAPK, Wnt, and other signaling pathways (72–74). Differentiated osteoblasts express bone sialic acid protein (BSP), a mineralized tissue-specific protein, which is probably involved in the early stage of bone mineralization (75, 76). IL-11 can promote the transcription of BSP directly by targeting CRE1, CRE2, HOX, and FRE in the proximal promoter of the BSP gene in rats (77). The effects of IL-11 on BSP transcription may be regulated by the transcription factors c-Fos, Dlx5, CREB1, JunD, c-Jun, Msx2, Smad1, and Runx2 (78). Furthermore, IL-11 promotes the transcription of the target genes of bone morphogenetic protein (BMP) through STAT3 (79), protein kinase A, PI3K, and ERK1/2 pathways (77). Recombinant human IL-11 (rhIL-11) induces the differentiation of osteoblasts in mouse mesenchymal progenitor cells, enhances the activity of alkaline phosphatase, and upregulates mRNA expression levels of BSP, osteocalcin, and parathyroid hormone (PTH) receptor (80). Moreover, rhIL-11 alone or together with recombinant human BMP-2 induces osteoblast differentiation into progenitor cells and accelerates bone formation without impairing bone strength (81–83).

As mentioned above, IL-11 can promote the transcription and expression of osteogenic factors through classical signaling pathways, induce osteogenic differentiation of mesenchymal stem cells, and promote normal bone development, bone remodeling and fracture healing. However, the mechanisms and pathways of IL-11 in regulating osteogenesis have not been fully elucidated. The effects of IL-11 on osteogenesis under normal and pathological conditions and related mechanisms need to be further studied.

### 3.2 Bone resorption

BM stromal cells produce IL-11, which induces osteoclast formation (84, 85) and directly affects osteoclast precursors (86). Because osteoblasts and mature osteoclasts express the mRNA of IL-11Rα, bone-resorbing cells as well as bone-forming cells are IL-11’s potential targets (12). *In vitro* studies have shown that IL-11 inhibits bone formation (87), triggers osteoclast formation by inducing osteoblast-mediated osteoid degradation and affects the development or survival of osteoclast progenitor cells (88). RANKL promotes osteoclast maturation and differentiation through the RANK/RANKL/OPG pathway, resulting in increased bone resorption (89). Independent of RANKL, IL-11 activates osteoclastogenesis through the JAK1/STAT3 signaling pathway and STAT3-mediated c-Myc expression (90, 91). Also, IL-11 inhibits osteoprotegerin (OPG) production, while the interaction between IL-11 and RANKL upregulates RANKL expression (92–95). Furthermore, IL-11 secretion by bone-derived endothelial cells was found to induce osteolysis in a murine calvarial bone organ culture model (96).

IL-11 is associated with osteolytic bone diseases. In rheumatoid arthritis (RA), a chronic immunological disease with bone destruction and synovitis, an association was observed between IL-11 expression and osteoarthritis (97–102). A previous study revealed that RA patients showed significantly higher serum IL-11 expression level than the control group (103). IL-11 controls bone turnover and is involved in bone loss induced by estrogen deficiency (86). Treatment of ovariectomized mice with IL-11 antagonists significantly increased cancellous bone volume, trabecular width, and osteoblast activity and decreased the number and activity of osteoclasts (104). Postmenopausal women who have osteoporosis show a significant increase in serum IL-11 levels, which were found to be positively correlated with the levels of bone resorption markers (NTX and DPyr). Serum IL-11 levels also accurately predicted bone turnover and the outcomes of antiresorption therapy (105). An *in vivo* study in estrogen-deficient rats showed that bone mineral density and osteocalcin concentrations were not significantly affected by rhIL-11 (106). Consistent with this finding, Sims et al. demonstrated that estrogen deficiency in mice affected bone homeostasis independent of IL-11 (86). Therefore, further investigations are essential to clarify the mechanisms underlying IL-11’s effects on osteoporosis.

**Figure 1:**
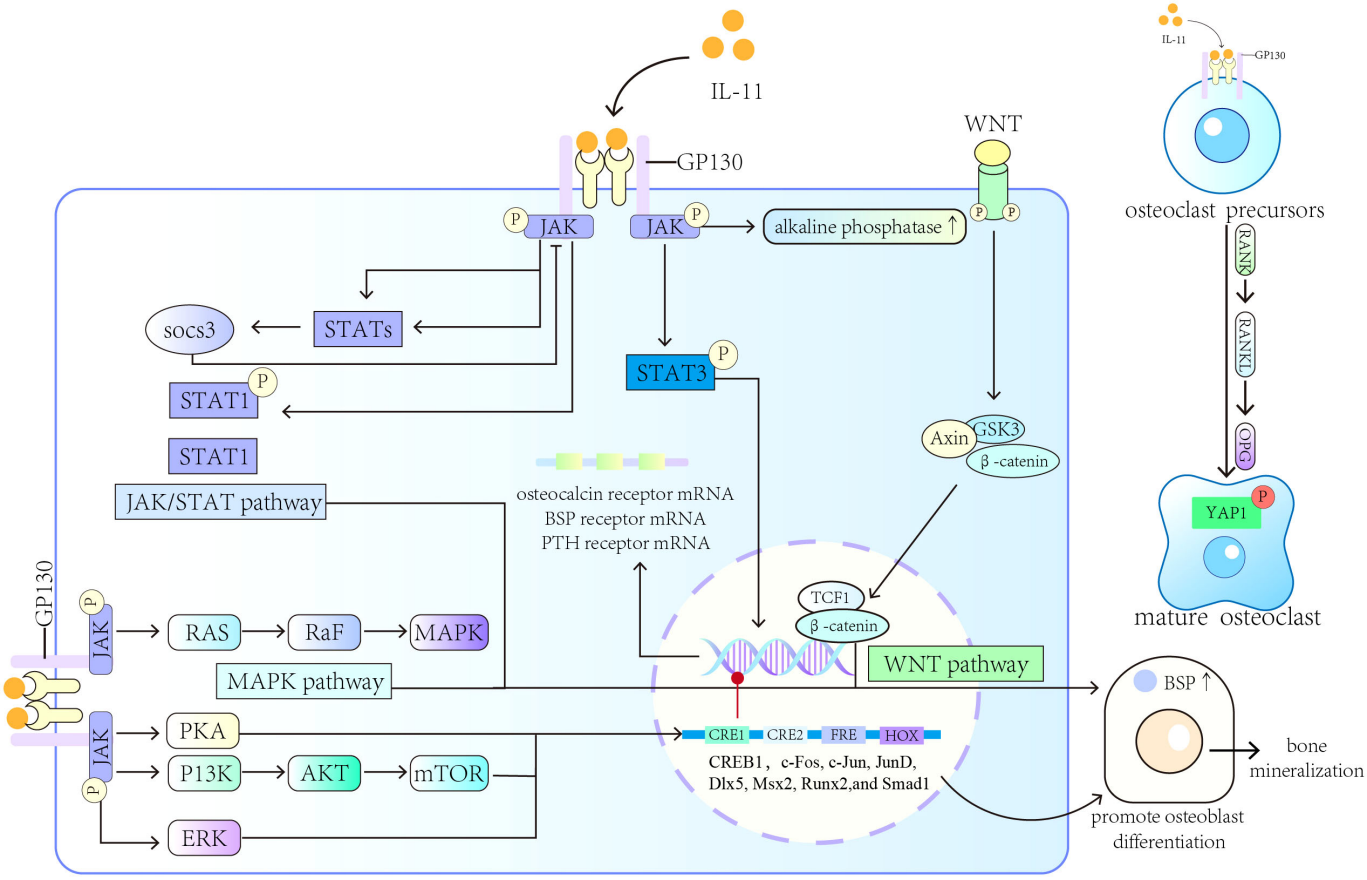
Roles of IL-11 in bone formation and bone resorption. IL-11 induces osteoblast differentiation and promotes the transcription of BSP through the MAPK, Wnt, and other signaling pathways. IL-11 can also induce osteoclast formation and different bone diseases.

In summary, IL-11 can induce osteoclast formation through Jak/STAT pathway, MAPK/PI3K pathway, phosphorylation of Yes-associated protein 1 (YAP1) and RANKL-independent mechanism, and is associated with rheumatoid arthritis, femoral arthritis, osteoporosis and other bone diseases. However, how IL-11 balances osteogenesis and osteoclastogenesis and its mechanism of action in bone diseases need to be further explored. A diagram of the main mechanism of the role of IL-11 in bone formation and bone resorption is provided in **Figure 1**.

### 3.3 Inhibition of adipogenesis

Both bone marrow adipocytes and osteoblasts are differentiated from bone marrow mesenchymal stem cells, expansion of bone marrow adipose tissue volume results in reduced osteogenic differentiation (107), the pro-adipose transcription factor PPAR promotes RANKL expression (108) and osteoclast formation (109, 110), and mature bone marrow adipocytes release pro-inflammatory cytokines (111); therefore, bone marrow adipose tissue is closely related to bone metabolism and bone remodeling. Established clinical studies have also shown that there is a negative correlation between bone marrow adipose volume and bone mineral density (112), and that estrogen deficiency, glucocorticoid use, and aging can cause expansion of bone marrow adipose tissue volume as the main pathogenesis of bone metabolic diseases such as osteoporosis (113, 114).

Many studies have investigated how IL-11 influences adipogenesis. Kawashima et al. performed DNA sequencing analysis (115) to determine whether adipogenesis inhibitory factor (AGIF) was associated with IL-11; the authors found that AGIF inhibited BM-derived preadipocyte production (116, 117). IL-11 decreased fat accumulation in the BM (79, 118), probably by preventing preadipocytes to differentiate into adipocytes (119). Furthermore, the formation of white adipose tissue and BM adipose tissue was significantly increased in mice following IL-11 knockout (72). Prostaglandin F2α activates STAT1 through an autocrine negative feedback loop mediated by IL-11/gp130 to inhibit adipocyte differentiation and adipogenesis (120, 121). IL-11 activates several signaling pathways in adipose tissue, including p44/42 MAP kinase, STAT1, PI3 kinase, and STAT3; promotes the migration and proliferation of adipose-derived mesenchymal stem cells (ADSCs) and skeletal myogenic cells; and attenuates ADSC apoptosis (122–124). IL-11 also regulates gene transcription and protein synthesis in differentiated adipocytes (125).

In summary, existing studies have shown that IL-11 can reduce bone marrow adipogenesis. However, the role and regulatory mechanism of IL-11 on bone marrow adipogenesis need to be further explored.

### 3.4 Hematopoiesis

Together with other cytokines, rhIL-11 enhances primitive hematopoietic and lymphopoietic progenitor cell formation in the BM (126). IL-11 promotes erythroid lineage formation and stimulates the proliferation and differentiation of megakaryocytes (127). Furthermore, in young mice, IL-11 increases the count of peripheral platelets based on the administered dose (128, 129). An *in vitro* study revealed that IL-11 synergizes with the Flt3 ligand, which is critically involved in early hematopoiesis in mouse BM (130), and this combination enhances megakaryocyte and progenitor cell production and stimulates the differentiation and expansion of myeloid lineage (131, 132). The autocrine regulator hepatocyte growth factor from myeloid stromal cells sustains the hematopoietic microenvironment by adhering to the ECM and promotes hematopoiesis by inducing IL-11 production through stromal cells (133–135).

IL-11-mediated effects on hematopoiesis could be clinically beneficial. The cytokine binds to the endothelial IL-11Rα and promotes angiogenesis induction, VEGF (proangiogenic factor) secretion, and fibroblast migration (99). Through the activation of the JAK/STAT3 pathway, IL-11 stimulates the secretion of stem cell factor (SCF), a c-kit ligand, in BM primitive stem cells (136, 137). IL-11 also reduced TGF-β1’s ability to suppress megakaryocyte production. IL-11 together with SCF or IL-3 promoted the recovery of leukocytes, platelets, and erythrocytes in mice subjected to irradiation (138). The effects of chemotherapy, radiotherapy, and BM transplantation on thrombocytopenia are reduced by IL-11 (139–142). Furthermore, IL-11 alleviates the function of the mitochondria by decreasing the production of reactive oxygen species, altering the mitochondrial membrane potential, maintaining mitochondrial number and function, reducing BM cell apoptosis, stimulating the growth of BM damaged by radiation, and minimizing radiation-induced immune response (143). A low-dose IL-11 therapy showed low toxicity and alleviated BM failure (144).

In summary, IL-11 can promote bone marrow hematopoiesis independently or in synergy with other hematopoietic factors, and can also protect mitochondrial function, which has a certain clinical therapeutic effect. However, extensive studies on IL-11 for clinical treatment of bone diseases are not enough. It is necessary to further explore the therapeutic effect, effectiveness and safety of IL-11 in different bone diseases.

### 3.5 Bone metastases

IL-11 participates in bone resorption induced by malignant osteolysis and tumor growth. IL-11 enhances chondrosarcoma cell migration by activating the NF-κB and PI3K/AKT pathways and increasing IL-11Rα-associated intercellular adhesion molecule-1 expression (145). Bone-derived TGF-β also acetylates KLF5, a transcription factor, in pancreatic cancer-related bone metastasis, and following its acetylation, KLF5 activates CXCR4 to induce osteoclast differentiation, thereby leading to IL-11 secretion (146). IL-11 also influences breast cancer (BC)-related bone metastases, and its expression is upregulated through the Smad2/3 and p38 MAPK pathway activation by the TGF-β/TGF-βR axis (147). Patients with BC and showing a high IL-11 expression are prone to develop bone metastases (148, 149). Smad4 induces IL-11 secretion (150) to enhance the morphological transition of epithelial cells to fibroblast-like cells (151). IL-11 also affects tumor cells present in the bone microenvironment to promote bone resorption and the formation of osteoclasts (152). Runx2/core-binding factor β (CBFβ) inhibits the Wnt signaling pathway by inducing sclerostin secretion and is required to modulate the functions of osteoblasts and osteoclasts in metastatic BC cells. IL-11 acts as a target gene for Runx2/CBFβ in metastatic BC cells and as a target of miR-124 (153) as it mediates miR-124’s inhibitory effect in osteoclasts and BC-related bone metastasis *in vivo (* 154).

IL-11 is an appropriate candidate for antimetastatic therapy (155). IL-11Rα also acts as a therapeutic target for bone metastasis (156, 157). Because IL-11 plays a mediating role in cancer-related bone metastasis, researchers developed a ligand-targeted peptidomimetic drug, namely BMTP-11 (bone metastasis-targeting peptidomimetic-11), which binds to IL-11Rα in the tumor vascular endothelium of castrate-resistant prostate cancer patients (158). Clinical trials demonstrated that BMTP-11 effectively treats human leukemia and lymphoma (159); moreover, as a cell surface target, it prevents the bone metastasis and growth of human prostate cancer and osteosarcoma (160). However, because of few clinical trials, the clinical application of BMTP-11 is limited, and its therapeutic effects on other cancers should be further investigated.

## 4 Regulators of IL-11

### 4.1 Bone formation

PTH and glucocorticoids modulate IL-11 expression to regulate the differentiation and apoptosis of osteoblasts (73). Recombinant human PTH (1-34) promotes osteogenesis by increasing Smad1/5 phosphorylation and ΔFosB expression to induce IL-11 gene transcription. Dexamethasone prevents PTH (1-34)-induced IL-11 gene transcription but does not affect ΔFosB expression and Smad1 phosphorylation (161–163). PTH can upregulate IL-11 expression to reverse glucocorticoid-induced osteoblast apoptosis (164, 165). Thyroid-stimulating hormone (TSH) modulates bone homeostasis by increasing the expression of IL-11 through the cAMP pathway (166).Senescence-accelerated osteoporotic SAMP6 mice showed a low IL-11 expression in BM cells in comparison with normal B6 mice of the same age (167). However, IL-11 secretion was increased significantly in human BM-derived mesenchymal stromal cells (168–170), probably through the cAMP/PKA pathway activation (171). Moreover, after BM cells isolated from normal allogeneic mice are introduced into the BM cavity of irradiated SAMP6 mice, the recipient mice showed substantial increment in IL-11 expression, bone mineral density, and trabecular bone density (172). Key transcription factors modulate the osteogenic effects of IL-11. For example, the heterodimer AP-1, i.e., activator protein 1, comprises c-Jun and c-Fos, and its reduced activity or synergy with the zinc finger protein Zac1 decreases IL-11 expression in BM stromal cells (173) and impairs bone formation in elderly people (174).

**Figure 2:**
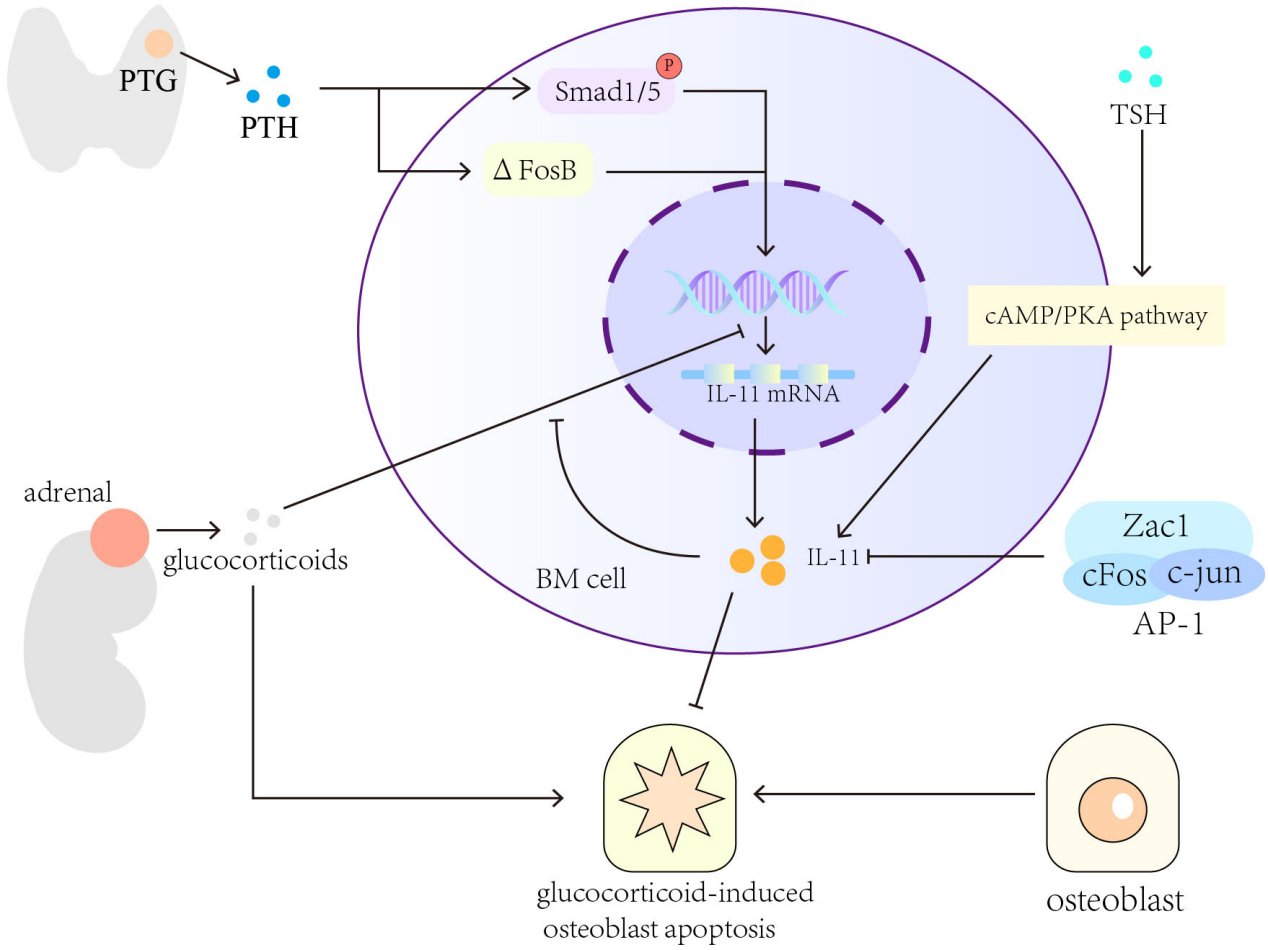
Cytokines and pathways that regulate the expression of IL-11 in bone formation. Hormones, for example, TSH, PTH and glucocorticoids, can regulate the expression and secretion of IL-11. Cytokines and cell aging will also affect the secretion of IL-11.

In summary, hormones, cytokines, and cell aging will affect the expression and secretion of IL-11. TSH and PTH can promote the expression of IL-11, while glucocorticoids, transcription factors (AP-1, ZAC1 protein), cell aging will inhibit the expression of IL-11 (**Figure 2**).

### 4.2 Bone resorption

Many cytokines are involved in regulating the role of IL-11 in bone resorption. IL-11 stimulates bone resorption through a mechanism involving PGE2 (175–177). Interferon-alpha downregulates IL-11 expression in human BM stromal cell cultures (178). TGF-β1 upregulates IL-11 gene expression by inducing the interaction between the transcriptional activators Runx2-c-Jun and Runx2-Smad (179). IL-11 participates in the process of bone resorption induced by lipopolysaccharides and enhances the formation of osteoclasts independent of IL-1 and TNF signaling *in vivo (* 180, 181). Heparin enhances the formation of the STAT3-DNA complex and transactivation induced by IL-11 as well as increases RANKL and gp130 activator expression (182). Treatment with heparin for a long term leads to cancellous bone loss in rats (183). Protease-activated receptor-2 expressed in osteoblasts inhibits RANKL expression, reduces RANKL to OPG expression ratio, and prevents IL-11-induced osteoclast differentiation (184). In rheumatoid joints, TNF-α and IL-1α synergistically stimulate IL-11 production through the production of PGE2, and indomethacin inhibits this synergistic effect (185). IL-4 protects against bone resorption by inhibiting IL-11 production in rheumatoid joints (186). Fas (Apo-1/CD95/TNFRSF6) ligand (FasL), a TNF superfamily member, is involved in the pathogenetic mechanisms of autoimmune diseases, including rheumatoid arthritis. In rheumatoid arthritis, FasL affects the expression of IL-11 in fibroblast-like synovial cells (187).

**Figure 3:**
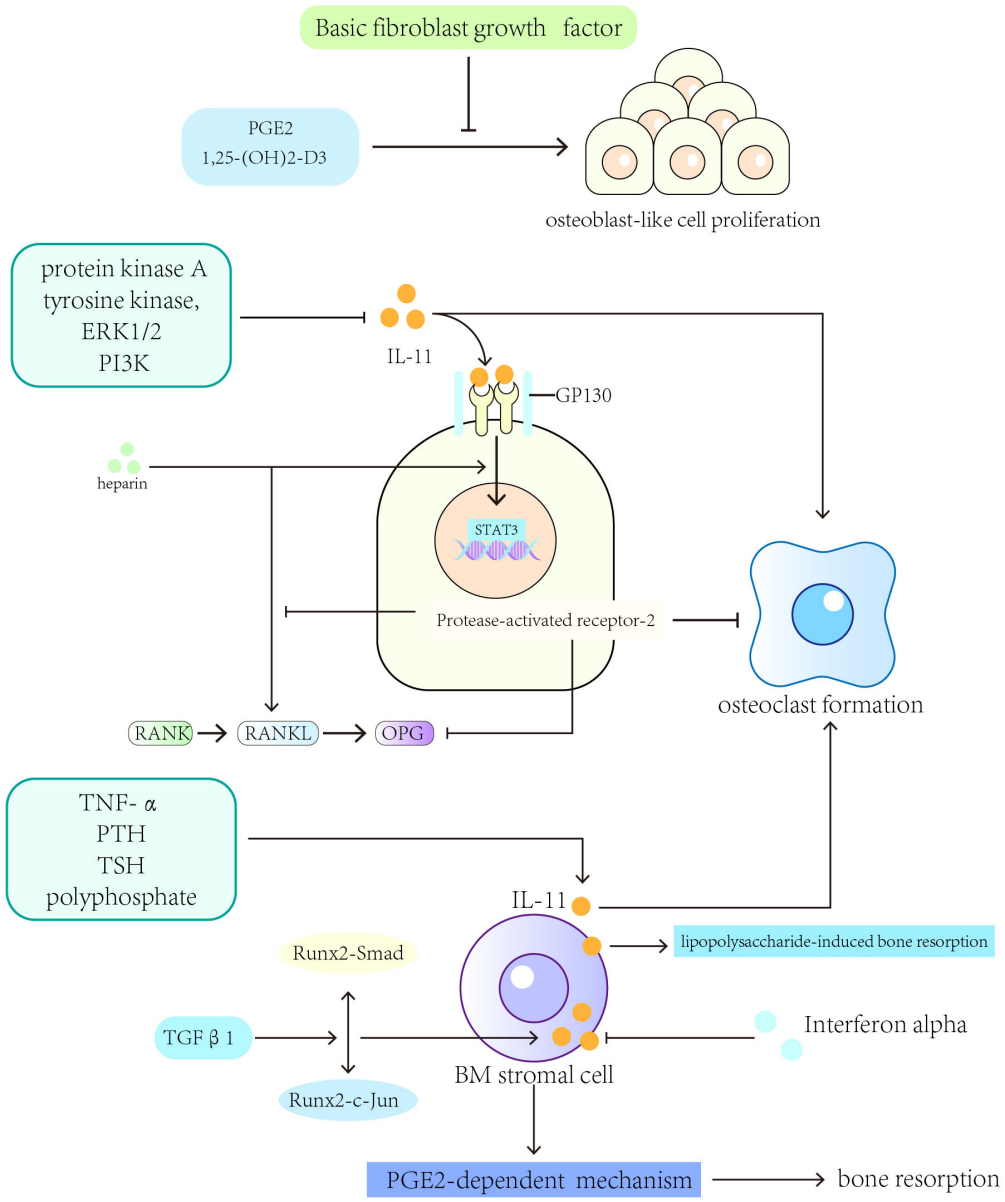
Cytokines and pathways that regulate the expression of IL-11 in bone resorption. IL-1, tumor necrosis factor (TNF)-α, PTH, TSH, TGF-β, polyphosphate, and prostaglandin E2 (PGE2) can regulate IL-11’s osteolytic activity.

In summary, several factors regulate IL-11’s osteolytic activity, including IL-1, tumor necrosis factor (TNF)-α, PTH, TSH, TGF-β, polyphosphate, and prostaglandin E2 (PGE2) (11, 12, 188–193) (**Figure 3**).

## 5 Limitations and further studies

The present study reviewed studies published in English over the past 21 years on how IL-11 affects bone metabolism and the regulators and signaling pathways that influence its effects. The results were consistent, although the analyzed studies were conducted in different countries.

The present study has some limitations. First, there were inconsistencies in cell type (human *vs*. mouse), tissue preparation (fresh vs. frozen), cell line origin, and gene expression analysis methods across different studies. Second, no statistical tests were performed.

Because IL-11 plays a variety of roles in bone formation and resorption, future studies should investigate the mechanisms and pathways through which IL-11 regulates bone remodeling and adipose tissue formation in normal conditions, the mechanisms and pathways of regulating bone metabolism in different pathological conditions (such as fracture, different types of osteoporosis, bone tumors, bone metastasis of malignant tumors, etc.), the cytokines and related mechanisms that affect IL-11 expression, the cytokines and related mechanisms regulated by IL-11 expression, the therapeutic potential of IL-11 to treat bone and joint diseases, and its safety and side effects as drug targets. At the same time, further research needs to focus on the relationship and difference between IL-11 and other inflammatory factors in regulating bone remodeling and bone metabolism.

## 6 Conclusion

IL-11 regulates inflammation and fibrosis, hematopoiesis, adipogenesis, fertility, and cancer development. Bone metabolism is the basis of physiological activities of bone tissue. Imbalance or pathological state of bone metabolism can lead to a series of bone diseases. The role of IL-11 in bone metabolism has been discovered earlier, mainly focusing on the regulation of osteogenesis and osteoclasis. In recent years, more research has focused on exploring the effect of IL-11 on bone metabolism. IL-11 promotes tooth and bone development and bone remodeling after fracture, induces osteogenic gene expression in differentiating osteoblasts, and improves mitochondrial function. The osteogenic effects of IL-11 are regulated by PTH/GC, cellular senescence and mechanical stress. IL-11’s bone resorption effects are facilitated by TNF-α, IL-1, TGF-β, 1,25-(OH)_2_-D3, PGE2, and other factors. Furthermore, IL-11 promotes bone resorption through the JAK/STAT and MAPK/PI3K pathways, YAP1 phosphorylation, and RANKL/OPG expression regulation. IL-11 is implicated in postmenopausal osteoporosis and RA. Although IL-11 has a dual role in regulating bone formation and bone resorption, the blockade of IL-11 signaling severely affects bone development and bone homeostasis. Recent studies have focused more on the central role of IL-11 in promoting bone formation and inhibiting bone marrow adipogenesis. However, overexpression of IL-11 may be associated with bone resorption-associated bone disease. Therefore, the regulatory role of IL-11 on osteogenesis and osteoclastogenesis needs to be further explored. IL-11 exerts its effects on ADSCs to inhibit white adipose tissue production. IL-11 also supports the formation of primitive hematopoietic and lymphopoietic progenitor cells in the BM; enhances the formation of peripheral erythrocytes, leucocytes, and platelets; and stimulates the differentiation and proliferation of megakaryocytes. The hematopoietic effects of IL-11 can be used clinically to treat bone and joint diseases. This study provides a basis to determine the underlying mechanism through which IL-11 exerts its effects and the signaling pathways participating in bone remodeling and to assess the therapeutic capability of IL-11 to treat rheumatic and osteoarticular diseases.

## Author contributions

YH: Conceptualization, Investigation, Writing – original draft. HG: Writing – review & editing. XG: Funding acquisition, Writing – review & editing. JL: Writing – review & editing. CB: Writing – review & editing. CH: Funding acquisition, Resources, Writing – review & editing.
